# Monkeypox: Some Keys to Understand This Emerging Disease

**DOI:** 10.3390/ani12172190

**Published:** 2022-08-25

**Authors:** Esperanza Gomez-Lucia

**Affiliations:** Department of Animal Health, Faculty of Veterinary Medicine, Complutense University of Madrid, 28040 Madrid, Spain; duato@ucm.es

**Keywords:** monkeypox, animals, epidemiology, transmission, diagnosis, treatment, genome

## Abstract

**Simple Summary:**

Monkeypox has entered our lives when we have not yet fully recovered from the SARS-CoV-2 pandemic. This close relative of the smallpox virus was restricted to tropical Africa, except on rare occasions when it caused human cases in the US, UK, Singapore, or Israel. Cases have recently been identified in multiple countries in at least the Americas, Europe, Asia and Australia. Fortunately, it produces much milder clinical symptoms and a much lower mortality rate than smallpox, but even so, we have learned not to overlook any possible pathogen, and there are many unknowns about the disease. What population groups does it affect? Could it cause a pandemic? What is the animal reservoir? Do animals suffer from monkeypox as much as humans?

**Abstract:**

In 1958, several monkeys in a Copenhagen laboratory developed a skin rash from which an orthopoxvirus could be isolated, which was named monkeypox virus (MPXV). However, the natural animal reservoir for MPXV is thought to be a rodent. The first human case occurred in 1970, and the incidence has increased progressively throughout the years. Starting May 2022, the number of cases outside Africa has soared, especially in Western Europe. There are two clades of MPXV, Congo Basin, with higher virulence and mortality, and Western Africa (WA). MPXV from the present outbreak has been proposed to be classified as Clade 3, distinct from the WA clade by at least 50 substitutions, which may increase human-to-human transmissibility. Most cases correspond to men in their 30s who have sex with men, and the possibility of sexual transmission is under investigation. Though there is no evidence of human-to-animal transmission, pets of positive human cases may be classified as low risk, including dogs, cats, and birds, who can be quarantined at home, and high risk, such as pet rabbits or mice, who should be isolated in official laboratories for observation. The current epidemiological data do not support the risk of a pandemic.

## 1. Introduction

Viruses are submicroscopic entities, with great simplicity, which have only either DNA or RNA, and for that reason, they must infect specific live cells in order to use their biosynthetic machinery to have more viral particles or virions made. Once this is accomplished, the new virions infect new cells. Some viruses are specific to their host and are said to have a narrow host range; on the contrary, others have a broad host range and can infect several species. Throughout history, viruses have produced many epidemics and pandemics in humans, animals, and plants [1,2]. One of such viruses has been smallpox virus, which originated in India or Egypt and spread throughout Africa, Asia, and Europe, leaving a trail of deaths behind. In the 20th century alone, a total of 300 million deaths have been attributed to this virus [3,4]. The mortality rate due to this virus has been an average of 30%, depending on the regions, the period, and the genetic conformation of the prevailing virus. Thanks to the geniality of a British doctor, Edward Jenner, the infection could be controlled to the point that it has been eradicated from Earth. He associated the lack of pock scars in the skin of milkmaids with the presence of pustules in the udder of cows and hypothesized that the latter could protect those in contact from contracting the disease. In 1797, he performed an experiment (which nowadays would not have passed any ethics committee) in which he practiced a scarification with the contents of the lesions in cows in a 12-year-old boy who did not contract smallpox when in close contact with infected persons [5]. This opened the door to the golden age of vaccinology, not lacking difficulties, such as how to bring the vaccine to other territories besides contiguous Europe, Asia, and Africa. This was solved by the Royal Philanthropic Expedition of the Vaccine, by which the Spanish doctor Balmis introduced the smallpox vaccine to these territories in 1803 [6], a first step to allow the WHO in 1979 to certify that the disease had been eradicated, a fact that was refrained in the WHO World Health Assembly in May 1980 [7]. Though undeniably a milestone in the history of mankind, its eradication has left an empty niche that might be filled by other viruses, such as the close relative monkeypox virus (MPXV), which produces a similar disease to smallpox, but a lower mortality rate.

In this review, the origins of monkeypox, how it was initially contained in Africa and has experienced a worldwide explosion, how it may be controlled by knowing the composition and genome of the virus, how it produces the disease in humans, and how it compares to cases in animals, and whether it may be of concern for domestic and wild animals are explored. It should be borne in mind that new data are constantly being published and hypotheses may change in view of new information.

## 2. Animal Hosts

The first known episode of monkeypox happened in 1958 in laboratory cynomolgus and rhesus monkeys imported to Copenhagen, Denmark, mostly from Singapore. Once in Denmark, they developed a vesicular disease from which a double-stranded DNA virus belonging to the genus *Orthopoxvirus* was isolated [8]. The virus was related to but distinct from smallpox virus (another orthopoxvirus) and was named monkeypox virus. Several similar outbreaks happened in 1959 and 1962 in different research facilities in the US [8]. A 1964 outbreak in a Rotterdam zoo sickened an array of animals, several of which died. Affected species included giant anteaters, orangutan, gorilla, chimpanzees, a gibbon, and a marmoset [8,9].

**Figure 1:**
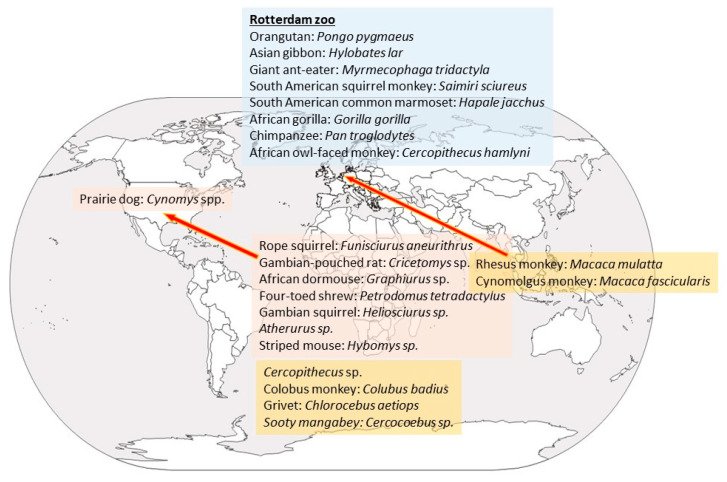
Species that have been found to be positive, either by virus isolation or presence of antibodies, and area of origin. Rodents are written in pink boxes; non-human primates in orange boxes and the species that were affected at the Rotterdam Zoo in a blue box.

With respect to wild animals, only six trapped in Africa so far have yielded the virus: three rope squirrels, a Gambian rat, a shrew, and a sooty mangabey monkey. Most of these animals were apparently healthy except for a squirrel (*Funisciurus anerythrus*) that was diseased [10]. Though viral presence in wild animals has been elusive, antibodies have been detected in more specimens, mostly in African squirrels, but also in *Cercopithecus* [9,11]. Thus, non-human primates seem to be only accidental hosts, and the natural host is likely a rodent or rodent related [12,13]. Several rodent species may be infected experimentally by MPXV, including rope squirrels (*Funisciurus* spp.), Gambian squirrel (*Heliosciurus* spp.), African dormice (*Graphiurus* spp.), Gambian giant pouched rat (*Cricetomys* spp.), and prairie dogs (*Cynomis* spp.) (Figure 1) [8]. In several of these species, the infection is asymptomatic, and more studies are needed to elucidate which is the natural reservoir and how the virus circulates in nature [8,14]. The ample variety of species that MPXV may affect confirms that, unlike smallpox virus, which only infected humans, MPXV has a broad host range, and no species should be overlooked as possibly involved in its epidemiological cycle.

## 3. Natural History of Monkeypox: Reported Outbreaks in Africa

The first human case was recorded on 1 September, 1970, in the town of Bukenda in the equatorial region of the Democratic Republic of Congo (DRC). It happened in a 9-month-old baby boy admitted to the Basankusu Hospital who had a vesicular eruption. The lesions would have passed unnoticed were it not that the WHO had established an intense surveillance of most of Africa to eradicate smallpox. In the DRC, the last smallpox case was diagnosed in 1971 [15], and smallpox vaccination had been interrupted nine months before the monkeypox episode. When the infection by MPXV was recognized, studies showed cases between October 1970 and May 1971 in Ivory Coast, Liberia, Sierra Leona, and Nigeria [16]. Since 1970 and throughout that decade sporadic cases were reported in the previous countries and in most of Equatorial Africa, including Cameroon, Gabon, Republic of Congo, Central African Republic, and South Sudan [17]. Very recently, Ghana has also been added to this list [18]. Studies show an escalation of monkeypox cases, especially in the highly endemic DRC, a spread to other countries, and a growing median age from young children to young adults. These findings may be related to the cessation of smallpox vaccination, which may have provided some cross-protection against monkeypox, leading to increased human-to-human transmission. As most cases have been happening in rural Africa, suspected underreporting may translate to an underestimation of the prevalence of MPXV [19].

The African countries that have reported the largest number of cases are the DRC (around 6000 cases), where it is considered to be endemic, and Nigeria (around 3000 cases). The number of cases has been gradually increasing since monkeypox was first reported in humans in 1970 [17]. Earlier epidemiological studies suggested that transmission of the virus between humans was not efficient and that most cases in Africa have happened due to repeated spillovers from animals [16,20,21]. Some of the reasons that favor an increased frequency of contact between animals and humans are deforestation, which exposes animals that are usually in the jungle; violence in the regions, which forces human groups to move to the more protected environment of the jungle; and lack of conventional food, so humans are compelled to consume other available sources, such as rodents, which might be infected [8]. The synanthropic rodent population, which increased in recent years in Africa, has led to more human-to-rodent interactions and thus increased transmission of MPXV [22]. All these factors are combined with the increase in human population in the affected areas and land shifts [2].

The number of cases in Nigeria has soared since September 2017, when an 11-year-old boy was diagnosed with monkeypox, starting an outbreak that is still ongoing [16,23]. Before 2017, the last confirmed case had been diagnosed in 1978. The WHO recognizes over 500 suspected cases and over 200 confirmed cases in this country, with an associated mortality rate of 3%. Epidemiological data suggest that the cases are not linked but that the virus has jumped several times from an animal host to humans [16]. The potential for accelerated adaptation to humans should be monitored through improved surveillance [20], and it has proven to be essential in view of the number of cases in Nigeria. Nearly 45 years after the end of routine smallpox vaccination, the larger and more interconnected immune-naïve population has crossed a threshold resulting in more sustainable human-to-human transmission of MPXV.

## 4. Monkeypox Outside Africa

The first time that monkeypox was detected outside Africa was in May 2003 in the USA. The first person outside of Africa to be diagnosed to have monkeypox was a 3-year-old girl bitten by one of her pet prairie dogs [9]. In two months’ time, 72 people attended the medical services because they had developed fever, vesicular eruption, respiratory distress, and lymphadenopathy (42 cases were confirmed [24]). The cases happened mostly in the Midwest and included the states of Illinois, Indiana, Kansas, Missouri, Ohio, Wisconsin, and New Jersey. No human deaths were registered due to this outbreak. The survey conducted on the first 53 cases showed that 50 had been in contact with prairie dogs (*Cynomis* spp.) kept as pets. The prairie dogs were traced back to an animal distributor in Texas, to which a number of African rodents had been imported from Ghana in April 2003. Upon analysis, 22 animals tested positive for MPXV, including Gambian pouched rats (*Cricetomys gambianus*), *Funisciurus* spp., *Heliosciurus* spp., *Cricetomys* spp., *Atherurus* spp., *Graphiurus* spp., and *Hybomys* spp. [25]. The genomic analysis of the virus from one person, a prairie dog, a rope squirrel (*Funisciurus* sp.), and a Gambian giant rat (*Cricetomys* sp.) showed that all four sequences were identical, confirming the epidemiological link. All prairie dogs were embargoed, and a ban on the import and breeding of African rodents was put into effect. This ban was partly modified in 2008 allowing US-born African rodents to be bred, but not imported [26]. The possibility of MPXV taking up permanent residence in wildlife outside of Africa, forming a reservoir that could lead to repeated human outbreaks is frightening [9], since this could imply recurring outbreaks in humans and the development of more dangerous virus variants. However, surveys of wild animals in Wisconsin and Illinois never found MPXV, none of the infected humans passed on the disease to other people, and worries about this exotic outbreak subsided [9].

In September 2018, the first human monkeypox cases exported from Africa were seen in the United Kingdom (*n* = 2) and Israel (*n* = 1) [27]. The case in the UK had a related nosocomial (health care worker) and a family home (an adult and a child in a household cluster) transmission events, becoming the first confirmed human-to-human monkeypox transmission events outside of Africa [28,29]. In May 2019, one case was detected in Singapore [30,31] and in December of the same year another case in the UK. They all corresponded to travelers who had been to Nigeria and developed the typical rash once back in their countries, and except for the nosocomial and household transmissions, all were unrelated. Isolates from all travelers and a Nigerian case shared a most recent common ancestor. Genetic variation for this cluster was lower than would be expected from a random sampling of genomes from this outbreak, but data did not support direct links between travelers [32]. Isolated cases were detected in 2021 in the UK and in Dallas (TX, Dallas, USA), as well as in travelers to Nigeria. The whole-genome sequencing of the Dallas case showed that the virus was consistent with a strain of MPXV known to circulate in Nigeria, but the specific source of the patient’s infection was not identified [33]. With the appearance of outbreaks beyond Africa, the global potential of the disease became evident [17].

Starting from 6 May, 2022, a large number of monkeypox cases not linked to travel to endemic countries was reported in at least 80 different countries (https://ourworldindata.org/monkeypox, accessed on 20 August 2022), mostly in Western Europe, but also in Central Europe, North America, in Australia. As of 18 August 2022, the total number of cases has been 44,500 cases, with a total of 5 deaths in non-endemic countries (https://www.cdc.gov/poxvirus/monkeypox/response/2022/world-map.html, accessed on 20 August 2022). The situation is evolving rapidly, and the majority of cases have been detected in Spain (with two deaths out of the 5792 cases), Germany, the UK, France, and Portugal, while numbers are also increasing fast in some other European countries, as well as in the USA and Brazil. Epidemiological data suggest person-to-person community transmission [34]. In addition, investigations into the early cases based on genomic data [35] suggest that the outbreak in Europe was certainly underway as early as mid-April and most likely earlier on [36].

## 5. Etiology: Genetic Types of Monkeypox Virus (MPXV) and the Phylogenetic Relationship to other Poxviruses

**Figure 2:**
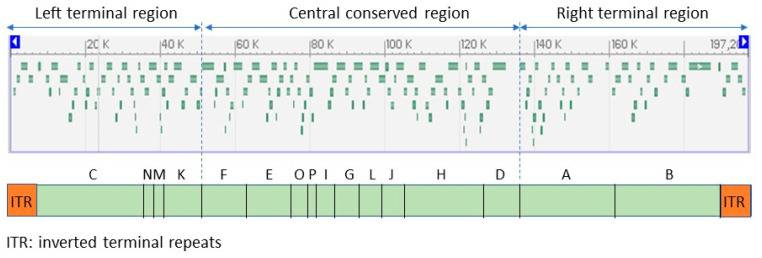
Open reading frames (ORF) identifiable in the monkeypox virus reference sequence (NC_063383; 197,209 bp) (**above**), and genomic sections in which they are organized (**below**). Adapted from GenBank and ViralZone.

Smallpox virus, vaccinia virus (the live virus component of vaccines against these viruses), and monkeypox virus are large double-stranded DNA. Like other poxviruses, MPXV is a brick-shaped particle of around 200 nm × 200 nm × 250 nm [27,37]. The virion is surrounded by a lipid envelope with distinct crests [38]. The genome is about 194 to 199 kbp and encodes about 200 proteins. It is a linear double-stranded DNA genome with covalently closed hairpin ends (no free 3′ or 5′ ends). Genes are closely packed and intergenic regions of more than 100 bp are rare. The central conserved region encodes “housekeeping” genes involved in transcription, replication, and virion assembly. The genes in the terminal regions encode proteins involved in host range and pathogenesis (Figure 2). Unlike other viruses that require cell receptors to infect a cell, some poxviruses have proteins on their surfaces that form a hydrophobic face, a water-repelling area that can bind nonspecifically to hydrophilic cell membranes and initiate the infection process [9]. This may be a reason for the broad host range of MPXV.

The genome structure is challenging. There are three main reasons for this: (a) it has a 6.5 kb inverted terminal repeats (ITR) at each end hard to resolve into individual copies with short read approaches, (b) it has a low G/C content and high poly A/T homopolymeric tracts that are hard to sequence accurately, and (c) there are local tandem repeats scattered across the genome [39,40].

Phylogenetic analysis has shown that there are two main distinct clades (recently proposed to be called lineages [35]). One is centered in the Democratic Republic of Congo and is called Central or Congo Basin (CB) clade or Clade 1. The other, called West African (WA) clade or Clade 2, lies mostly between the Equator and South of the Sahara. Cameroon is the only country where both clades have been detected to co-exist. The CB clade produces more severe cases and is more contagious than the WA clade and is associated with 10.6% (95% CI: 8.4–13.3%) mortality vs. 3.6% (95% CI: 1.7–6.8%) estimated for the WA clade [17,23,41].

There is ongoing research to determine precisely which genes are responsible for the higher virulence and transmissibility of the CB clade, compared with the WA clade. This is proving difficult due to the large size of the genome and because relatively few sequences are available in Africa due to economic constraints [42]. The Portuguese researcher Palacios suggested that one of the differences between both clades might be the N2R and N3R deletion observed in the CB clade, not present in the WA clade, and which is associated with human-to-human transmission [20].

The recent MPXV outbreak has triggered intense studies. Shotgun metagenomics allowed the rapid reconstruction and phylogenomic characterization of the first genome sequences of the MPXV outbreak variants, placing them in Clade 3 (within the formerly designated West African clade, which also includes Clade 2) [35]. This Clade 3 comprises human MPXV (hMPXV)-1A, with newly classified lineages A.1 and B.1. Lineage B.1 includes all MPXV genomes from the 2022 outbreak. It was estimated that the B.1 lineage of this clade emerged in Europe in February 2022 [43].

Although researchers need more data to confirm their hypotheses, the sequences of the recent cases they have evaluated so far are nearly identical to each other, suggesting that a thorough epidemiological investigation might find that the recent outbreaks outside Africa all link back to a single origin. A second possibility is that the virus may have been introduced through earlier outbreaks and circulated unnoticed outside Africa in human or animal populations; an argument against this possibility is that monkeypox virus was known to produce visible local or generalized lesions in most infected humans and would hardly have passed unnoticed [42]. However, in the present outbreak, WHO has warned that the typical lesions may be absent or difficult to identify, which would have allowed the spread of the infection.

Regardless of the genomic uniformity, results show that the virus in the present outbreak is rapidly mutating, especially for a DNA virus [35], and which is a sign of MPXV microevolution during person-to-person transmission, suggesting an adaptation to the human host [44]. Around 50 single-nucleotide polymorphisms (SNPs) (or substitutions) have been identified scattered across the genome when compared to the closest relatives, those of the European cases of 2018 and 2019 [35], which likely represent a recent evolutionary jump. Up till now, only 1 or 2 substitutions/site/year had been detected [45], suggesting that 50 changes could represent an accelerated evolution of the virus [35]. Many of the mutations are silent, and they do not change the encoded amino acids, but at least 21 mutations are non-synonymous. Some affect proteins involved in human transmission, virulence, or interaction with antiviral drugs. It is difficult to recognize the impact of these substitutions, which would require structural modeling, but when compared to other orthopoxviruses, an approximation may be predicted. Of the non-silent mutations, there are three that are considered high priority, all in the B1/B2 proteins: D209N, P722S, and M1741I. This hypermutation signature suggests the potential action of host enzymes, such as Apolipoprotein B mRNA Editing Catalytic Polypeptide-like (APOBEC)-3 in viral evolution. APOBEC molecules form part of the innate immune system. These enzymes may edit the viral DNA and block viral replication [46,47]. They inhibit many viruses by introducing mutations. Occasionally, they do not completely inhibit viral replication, but allow the expansion of hypermutated variants, edited by the enzymes, but which are viable and with similar characteristics to the parental strains, which may escape the immune response [44]. This could be the case of these B1/B2 mutations, which could be an important target for antibodies, and they could allow escaping from the protection conferred by vaccines [48]. B1/B2 is a T-cell inhibitor also found in cowpox virus, camelpox virus, and horsepox virus. When the protein is knocked-in into non-virulent cowpox virus, the resulting virus increases disease severity and mortality in rats [39].

## 6. Epidemiology

In Africa, the median age at presentation has increased from 4 (in the 1970s) to 21 years (2010–2019) [17]. In the current 2022 outbreak in non-endemic countries, most of the cases have been detected in males between 18 and 50 years. As of 4 July 2022, 21 cases of the 7553 had been reported in women. The risk for children is considered low [49]. The ongoing epidemic differs from previous outbreaks in terms of age (more than half of the individuals have been in their thirties, which may be associated with a lack of smallpox vaccination in their childhood), sex/gender (most cases being males; for example, as for 24 June, of the 528 cases diagnosed by PCR by an international collaborative group of clinicians, 98% were men, with an average age of 38 years), risk factors, and transmission route, with sexual transmission being highly likely [50]. In addition, most cases have been neither part of identified transmission chains, nor linked to travel or had contact with symptomatic persons or with animals, suggesting the possible previously undetected spread of monkeypox [51], which may have gone undetected for quite a while [52], amplified by recent large social events and increased travel. According to WHO, up-till-now few cases required hospitalization (in Spain, as of 16 June, of the 497 cases, 11 required hospitalization), and as of 18 August, 5 deaths have been registered (2 in Spain, 1 in Brazil, 1 in Peru, and 1 in India) (https://www.cdc.gov/poxvirus/monkeypox/response/2022/world-map.html, accessed on 20 August 2022). As the data are evolving rapidly, the basic and effective reproduction rates (R0 and Re) in different populations have not been established [22].

Preliminary risk factors in the ongoing epidemic are: being a young male; having sex with other men; engaging in risky sexual behaviors and activities, including condomless sex, multiple sexual partners, and human immunodeficiency virus infection [53,54,55,56]; and a story of previous sexually transmitted infections, including syphilis [53,56]. In a Spanish study, 54% of the patients had had a positive diagnosis of sexually transmitted infections (STIs) in the previous months, 42% were positive for HIV, and 76% had a concomitant STI at the time of diagnosis of MPXV [56]. 

## 7. Transmission

The specific mechanism for transmission is not well established and still leaves many questions unanswered. However, the data from other poxviruses suggest that the virus is present both in the skin lesions and in droplets exhaled while breathing.

An infected animal may transmit MPXV to a human through direct or indirect contact with its blood, body fluids, or lesions, when the animal is managed or succeeds in biting or scratching the person. Within humans, the virus may be transmitted through respiratory droplets (>5 mm, which makes them heavy and thus cannot travel long distances, requiring close contact), direct or indirect contact with body fluids, content of the lesions, or recently contaminated surfaces or materials. The virus penetrates through the injured skin, respiratory tract, and mucous membranes, such as the eyes, nose, or mouth. It may cross the placenta and produce miscarriages in the first trimester and fetal death, with the stillborn showing typical skin lesions [57,58]. Newborns may also contract the infection during delivery or soon after birth [59].

The possibility of sexual transmission is raising big concern, as a large number of cases have happened in men who have sex with men (MSM) [34,36,50,54,56,60,61,62]. The development of skin lesions and eruptions in the genital and perianal regions suggests that direct physical contact with lesions during sexual contact is a likely route of transmission [52]. The temporal association observed between sexual intercourse, increased inguinal lymphadenopathy, and recurrence of rash suggests a possible genital reservoir for MPXV, which needs to be further studied. Though biological samples from the seminal fluid were positive for MPXV viral DNA in some studies [61,63,64], another study found no MPXV in semen [28]. However, it would be advisable to recommend men to use condoms for their sexual relationships up to 12 weeks after the complete recovery from the infection until more is known about levels of the virus and potential infectivity in semen during the period that follows recovery (https://www.who.int/news-room/questions-and-answers/item/monkeypox, accessed on 20 August 2022).

## 8. Pathogenesis

Like most other poxviruses, MPXV has a tropism for epithelial cells and, to a lesser degree, for macrophages and fibroblasts. These viruses typically infect the stratum spinosum of the dermis. In cases prior to the present outbreak, the lesion starts with a macule, which is mostly an unraised reddish area of the skin, followed by a papule, in which the skin starts to raise. The following stage is a vesicle, characterized by the presence of clear liquid in its inside. The liquid quickly turns yellowish, characteristic of a pustule. The surface of the lesion progressively thins out and weakens and finally opens to free the content, full of infective viral particles, and forms a crust that eventually falls. However, in the present multi-country outbreak, dermatologists are reporting that the lesions are pseudopustular, with whitish solid content, a necrotic center, and an erythematous hale, which evolve to a more purulent, necrotized, and even ulcerative aspect [56]. As the lesion originated deep in the dermis, usually scars of pockmarks are left as a result of the infection [38,65,66,67]. The lesions happen in the outer skin, as well as the accessible mucosae, such as the oral or anal mucosa, but also in the epithelial cells lining the internal organs [68].

## 9. Symptoms in Humans

**Figure 3:**
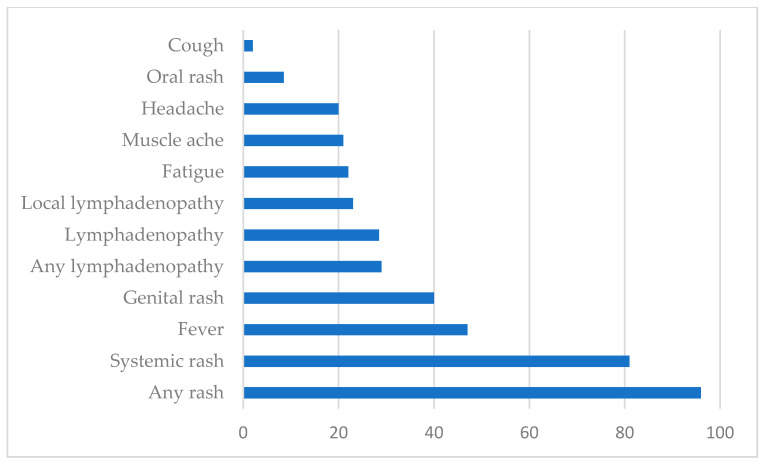
Signs and symptoms among confirmed cases of monkeypox (showed in percentage of presentation). Globally, as of 8 July 2022 [73].

After an incubation period lasting 6 to 13 days (but which may range from 4 to 21 days), the WHO differentiates two stages. In the first stage, which may last up to 5 days, the patient may suffer (numbers in parenthesis correspond to the present 2022 outbreak): fever (54% of the patients), intense headache (32%), backache, and myalgia (44%), as well as severe asthenia (44%). Lymphadenopathy (lymph nodes of the neck, axilla, and groin) is present in a large percentage of affected persons (56%) (Figure 3) [23,50,56,59,69,70,71]. In the second stage, which starts 1 to 3 days after the onset of fever, an exanthema, the typical poxviral lesions described above, develops. The lesions are itchy and can be very painful, and while typically in the previous outbreaks were seen mostly in the face (95%), the limbs, mostly in the hands and feet (75%), and the oral mucosa (70%), in the present 2022 outbreak, they are mostly in the genitalia or perineal region (Table 1) [50,56,60,61,72]. Lesions in the conjunctiva are less frequent (20%) but can lead to sight-threatening keratitis [25,28]. However, in the ongoing epidemic, the clinical presentation is atypical and unusual, being characterized by anogenital lesions and rashes that relatively spare the face and extremities [53,54]. After a localized beginning, the lesions usually spread to the rest of the body [50,56]. The development of whitlows (inflammation of the fingertips) and proctitis (inflammation of the rectal mucosa) may be seen [50,56]. The process is self-limiting within 4–6 weeks, and only around 2% of the cases need to be hospitalized, mostly to control pain and preventive vigilance of symptoms such as severe dysphagia, conjunctivitis, or suspected perforation. A person is infective from the beginning of the symptoms until all the ulcers have healed up and a new layer of skin has formed, which may take several weeks.

Severity depends on several factors, including age (more severe in children), health (more severe in immunocompromised individuals, in whom the prognosis is worse and has a higher mortality risk), and the development of complications. Complications may include secondary infections due to bacterial colonization of the open lesions; septicemia, from a systemic spread of secondary bacteria [72]; bronchopneumonia, as a frequent entry route is the respiratory route; and corneal infection, which may lead to blindness [74]. Mortality historically ranges from 0% to 11%, and in the outbreak which started in 2017 in Nigeria, it has been calculated to be 3–4.7%. It is higher in children.

## 10. Diagnosis and Treatment

As with all transmissible diseases, the sooner diagnosis is achieved, the sooner close contacts can be located and isolated, which will decrease viral transmission. Presumptive identification of monkeypox based on clinical symptoms is important for identification of suspected cases during surveillance, but confirmation must be achieved by laboratory diagnosis. The selected methods are molecular techniques, if possible real-time PCR (rt-PCR), preferably in biosafety level-three facilities. rt-PCR targets conserved regions of extracellular envelope protein gene (*B6R*), DNA polymerase gene (*E9L*), or DNA-dependent RNA-polymerase subunit 18 (*rpo18*) [16,75]. Whole-genome sequencing may be achieved relatively fast using next-generation sequencing (NGS) [20,35,76,77], but the technology is expensive, and processing of data obtained requires huge computation power. Fast and portable point-of-care methods (POC) may be considered in endemic countries, and their sensitivity and specificity should be evaluated for the present outbreak. These include the GeneXpert MPX/OPX (which targets the *E9L* gene, with 98.8% sensitivity and 100% specificity [78]), the loop-mediated-isothermal-amplification (LAMP, which targets genes *D14L* and *ATI*, with a sensitivity of 72–80% and a specificity of 100% [79]), and the recombinase polymerase amplification assay (RPA, which targets the *G2R* gene, with a sensitivity of 95% and a specificity of 100% [80]).

Antibody detection may render false positive results due to the possible presence of antibodies against vaccinia, the orthopoxvirus used for vaccinating against smallpox, though specific IgM may suggest recent MPXV infection, even in previously vaccinated individuals [16]. Electron microscopy and tissue culture have also been used for diagnostic purposes [27,81]. MPXV grows in Vero cells, where it produces a cytopathic effect consisting of cell detachment and rounding, with evident viral factories [27].

MPX may be clinically mistaken for chickenpox, a disease produced by the varicella-zoster herpesvirus. This disease is characterized by the development of itchy raised pink or red papules, small fluid-filled vesicles that break and leak, which eventually are covered by crusts and scabs. Usually, there is no lymphadenopathy. The molecular tests mentioned above are able to discriminate one infection from the other [82].

Several molecules, studied to fight smallpox, have been tested for MPXV, including mitoxantrone [83], cidofovir, tecovirimat, resveratrol, ribavirin, neutralizing antibodies, and small interfering RNA [84]. They have shown to have an effective anti-MPXV activity in vitro and in vivo. The only antiviral authorized by the European Medicines Agency (EMA) for the treatment of monkeypox is tecovirimat, approved in January 2022 under exceptional circumstances [85]. Tecovirimat targets the membrane protein VP37 of vaccinia virus required for the production of extracellular forms of virus. Another antiviral with activity against orthopoxviruses is brincidofovir (CMX001 or hexadecyloxypropyl-cidofovir). This antiviral has been approved by FDA in June 2021 under the agency’s Animal Rule (https://www.fda.gov/drugs/news-events-human-drugs/fda-approves-drug-treat-smallpox; accessed 20 August 2022). It is an orthopoxvirus nucleotide analog DNA polymerase inhibitor. Brincidofovir (200 mg once a week orally) is not devoid of adverse effects, being the most common diarrhea, nausea, and vomiting, as well as elevation in hepatic transaminases, which may require interrupting therapy [28,33]. Tecovirimat (200 mg twice daily for 2 weeks orally) appears to be better tolerated and adverse effects are mild (headache and nausea) or non-existent while shortening the duration of viral shedding and illness [28,86]. Another antiviral evaluated has been isatin beta-thiosemicarbazone (IBT), but it seems less effective against MPXV than against vaccinia virus, as less dsRNA, the trigger for the effect of this drug, is produced in the MPXV infection [87]. Cidofovir, authorized at the EU level for the treatment of cytomegalovirus retinitis in patients with AIDS and normal renal function, has proven activity against poxviruses in in vitro and animal studies. It has marked associated nephrotoxicity [84]. Antiviral treatment may require antibiotic complementation if secondary infections develop.

## 11. Prevention through Vaccination

The timing of the cases and the age of the patients suggested from the early 1970s that smallpox vaccine (the vaccinia virus) could provide an 85% protection from monkeypox and decrease the severity of the disease, and certain smallpox vaccines, such as Imvanex, a third-generation replication-deficient Modified Vaccinia Ankara (MVA), have been licensed by the EMA since 2019 for use to prevent monkeypox [85]. In animal models (non-human primates), it has shown protection against severe disease after lethal challenge with MPXV and comparable immune responses to traditional smallpox vaccines (significant prevention of morbidity and mortality compared to non-vaccinated animals). One study demonstrated non-inferior responses compared to licensed ACAM2000 (second-generation live smallpox vaccine). Unlike some vaccines against COVID-19, which may take up to two weeks after a second dose for optimal protection, smallpox vaccines are thought to protect against monkeypox infection if administered within four days of exposure, due to the long incubation period of the disease [42].

As there is a scarcity of vaccines, use must be prioritized. First-line users should be health care personnel, including doctors and nurses and other health personnel in hospitals and health centers, as well as veterinarians. In addition, the ECDC recommends prioritizing groups of men who have sex with men (MSM) at higher risk of exposure, along with the front-line staff with a risk for occupational exposure, in developing vaccination strategies [49]. Because orthopoxvirus infections resulting from occupational exposures can be severe, the Advisory Committee on Immunization Practices (ACIP) has continued to recommend preexposure vaccination for these workers since 1980 [88], when smallpox was eradicated. The vaccines recommended by the ACIP are ACAM2000 and JYNNEOS, both live replication-defective vaccinia virus vaccines, for preexposure vaccination of persons at risk for occupational exposure to orthopoxviruses [88].

Ring vaccination may be used to contain outbreaks, vaccinating close contacts of positive cases. However, due to the limited availability of the vaccine, a community effort should be encouraged for early detection of new cases and intensive observation of close contacts (for 21 days, the length of the incubation period) to break the transmission chain [49].

## 12. Animal Models

Animal models proposed to study monkeypox pathogenesis include non-human primates (such as *Macaca fascicularis* and *Macaca mulatta*), rodents (including *Graphiurus* and *Cynomis*), and transgenic mice (knock-out STAT1 mice). Susceptibility in animals varies depending on the infection route and their age. Lesions may present as skin or epithelial eruptions, which may evolve into purulent lesions. Disease features included viraemia, prolonged monkeypox virus DNA detection in upper respiratory tract swabs, reactive low mood, and monkeypox virus PCR-positive deep tissue abscess [89]. Besides their use to study pathogenesis, animal models have also been used to evaluate the effectivity of antivirals and vaccines [8].

## 13. Clinical Signs in Animals

Natural infection in animals, especially in non-human primates, resembles the clinical signs observed in humans. *Macaca fascicularis* and *Macaca mulatta*, kept in research centers in different countries, developed vesiculopustular skin eruptions over the entire trunk, tail, face, limbs, palms of the hands, and soles of the feet. Most times, the lesions formed crusts, healed, and fell off, leaving a scar [8]. In other instances, severe facial and cervical edema, hemorrhagic ulcerations, dyspnea, and bloody diarrhea have developed, and the animals have died [8].

The first case of natural infection in a dog was described in August 2022 [90]. The dog, a 4-year-old male Italian greyhound with no medical issues, had contracted the infection from his owners, two men that had had multiple sexual relationships with other men six days before they developed anal ulceration. The dog slept with his care-givers. Twelve days after the symptoms started in the men, the dog developed mucocutaneous lesions, mostly pustules in the abdomen and a fine anal ulceration, which was positive for MPXV by rt-PCR. This means that MPXV produced a disease in the dog that did not act as a vector for it. The sequence of the virus from the dog and the men was confirmed by next-generation sequencing (NGS) to be human MPXV-1 (hMPXV-1), Lineage B.1, which is the one circulating in the present multi-country outbreak. The homology of both genomes in the 19.5 kbp sequence compared was 100% [90].

Experimental infections have been carried out in primates, such as Cynomolgus monkeys (*Macaca fascicularis*, mostly through aerosolized route) [91,92,93,94], and rodents, including Gambian giant rat (*Cricetomys gambianus*) [14], rope squirrels (*Funisciurus* spp.) [95], ground squirrels (*Spermophilus* spp.) [96], African dormice (*Graphiurus* spp.) [97], and prairie dogs (*Cynomis* spp.) [98,99]. Some of these studies have been undertaken to assess the efficacy of different drugs [99,100,101,102] or vaccines [103,104,105] towards MPXV and infection. The disease seems to be more severe in non-human primates (though it might depend on the challenge dose, but not exclusively) [91], and many experimental animals die or need to be euthanized [94].

Lesions in experimentally infected animals, especially in non-human primates, closely resemble those seen in humans. In cynomolgus monkeys (*Macaca fascicularis*) fever developed 2–6 days after virus inoculation, palpable lymphadenopathy (which differentiates monkeypox from smallpox also in monkeys) at Days 3–4, and papovesiculopustular skin lesions on Days 4–6. Skin pocks first became visible on the face, oral mucosa, and the axillary and inguinal regions, and these then became generalized, with an estimated 1000 lesions present at the time of death [106]. Viremia and viral loads in other locations peaked around Day 10, and in survivors, these parameters decreased until Day 28 post-exposure [91]. The respiratory organs have been seen to be also severely affected [93]. The disease in rodents is similar, though they may be more resistant to the virus, and many recover from the experimental infection. Skin vesiculopapular lesions develop mostly in the oral cavity, including the tongue and gums [14]. In prairie dogs, in vivo imaging showed the presence of MPXV in the nose, lymph nodes, intestines, heart, lung, kidneys, and liver even before the appearance of skin lesions [107]. Rabbits have been found to be quite susceptible to the infection by MPXV. When they were challenged by different routes, both adult and 10-day-old rabbits developed clinical signs, including fever and rash, loss of weight, and adynamia. Mortality was around 85% in the 10-day-old rabbits [108]. The same was true for white mice, which experienced weight loss and adynamia when inoculated with MPXV. Mortality ranged between 24 and 100% [108]. For a comprehensive review of monkeypox infections in animals, see [8].

## 14. Monkeypox, Pets and Veterinarians

Undoubtedly and understandably, there is big concern that patients may transmit the virus to their pets, which may perpetuate and maybe even transmit it to wild animals, starting a new animal reservoir in non-endemic countries. The risk also exists that home pets (as well as farmed rabbits) could transmit MPXV to humans, and many pet owners may consult the veterinarian about what to do. Public health officials in several countries have advised people who have monkeypox lesions to avoid contact with their pets [9]. Pets from infected persons need to be confined to confirm that they are MPXV-free. Pets may be classified into two categories: low-risk and high-risk transmissors. The first category included dogs, cats, and birds. However, since the first case of MPX in a dog transmitted from their owners [90], this species may be re-evaluated and removed from this category. As these species have not been reported to be infected by MPXV, they may be isolated at home at least for 21 days after the diagnosis of the human-positive contact, but owners are advised not to take them for walks (especially by the MPXV-infected person), let them roam, or other activities that involve leaving the housing premises. If dogs need to be walked, they should be with a muzzle and on the leash, avoiding contact with other animals. In all cases, they should be kept separate from their infected owners and not allowed to sleep with them, which was the case of the infected dog reported by Seang et al. [90]. Though cats are susceptible to cowpox virus and to zoonotic *Orthopoxvirus*, which can be transmitted to humans, not a single case of monkeypox attributable to a cat has been reported in Africa, and a serological survey failed to identify any seropositive cats [24]. In regard to ferrets, there are no data available, and they have not been found to be infected by *Poxviridae*. However, following the principle of caution a high-protection mask (FFP2, KN95, or similar) and latex or vinyl gloves should be used when changing the bedding or the sandbox, preferably by smallpox-vaccinated individuals.

The second group includes pet rodents, such as hamsters, guinea pigs, mice, rats, gerbils, or rabbits. In this case, as they have been shown to be susceptible to the virus, isolation and quarantine should be done in government facilities for a better and more monitored observation. As the last resort, they should be euthanized [109]. Adult albino rabbits have been shown to be highly susceptible through “natural” routes of infection. Even though rats (*Rattus norvegicus*) and pet mice (*Mus musculus domesticus*) have not been shown to be susceptible by “natural” routes of infection, under experimental conditions, the neonates of these species are highly susceptible to MPXV [8]. Both rats and mice have synanthropic relatives, unwelcomed companions in the cities and other human settings, which may raid trash and could come contaminated with infected waste [9]. Thus, residues from rodents and other pets need to be sprayed with home disinfectants, such as chlorine bleach, and introduced in hermetically closed bags.

If an animal presents compatible MPX clinical signs, the Official Veterinary Services must be contacted immediately. The lesions usually start in the head and later spread to the rest of the body, mostly to the limbs and ears. The inflammation is followed by crusting and the animal may develop fever, anorexia, changes in its behavior, and lethargy. Young or immunocompromised cats may have a severe respiratory disease (pneumonia), which may be fatal. Veterinarians should keep in mind a number of measures to minimize the potential transmission of the virus to other animals and to humans (Table 2, [110]). Samples for diagnosis include skin lesions, vesicular fluid, smears of vesicles, exudates, scabs, or swabs from the oral cavity.

So far, human-to-rodent transmission has not been proven. However, the risk exists that the virus goes back to animals and that it finds an animal reservoir in non-African settings [8,24]. Since African squirrels may play an important role as a reservoir of MPXV, the potential infection of wild squirrels in North America and Europe should be taken into account.

## 15. Analysis of the Infection by Monkeypox under the Concept of One Health and Conclusions

As of 23 July 2022, and in view of the big soar of MPX cases, the WHO has classified the present outbreak as a Public Health Emergency of International Concern (PHEIC), recognizing the complexities and uncertainties associated with this public health event. A PHEIC is the highest alert degree, which up till now was only applied to COVID-19 and polio. The identification of the very likely transmission of infected humans to a dog has opened many disturbing concerns, and surveillance and measures need to be intensified to avoid domestic and farmed animals from getting infected and spreading the virus to rodents, allowing MPXV to become endemic. Measures should also include training health workers and veterinarians to correctly assess their patients and clients. It is clear that the understanding of monkeypox still has many gray areas: the animal reservoir is not identified, the risk factors for zoonotic and human-to-human transmission, and the ecological conditions favorable to its emergence are not fully described. To respond to these questions, a combined effort of different experts must be put into effect, and this needs to include human health doctors, veterinarians, biologists, ecologists, mathematicians, epidemiologists and any other specialist who may shed light on the problem [111]. The possibility of MPXV infecting some rodent species in non-endemic countries is alarming and needs to be urgently addressed. The spread of the infection may be hindered if the correct strategies are put into effect in communities with high exposure risk, avoiding the stigmatization of the main affected groups. We are still in time to control the present outbreak and avoid the possibility that MPXV-infected humans spread it to wildlife outside Africa. Let us not miss this opportunity. It may be the last one.

## Figures and Tables

**Table 1:** Main differences reported in the clinical presentations of monkeypox in patients from the present outbreak and from previous outbreaks. Symptoms in one column do not mean that they cannot be present in the other. However, the frequency of presentation is lower.

2022 Outbreak	Previous (African) Outbreaks
Lesions in genital (penis, testicles, labia and vagina) or perineal/perianal area (anus), which may not spread further	Extensive characteristic centrifugal rash, starting from the site of primary infection, which rapidly spreads to other locations, characteristically palms and soles
Low number of lesions (a few, a single, absence)	Fever
Anal pain and bleeding	Swollen lymph nodes
Asynchronous development of lesions, which may be in different stages	
Possible absence of prodromal period, with no fever, malaise or other constitutional symptoms	

Adapted from [18].

**Table 2:** Measures to be observed by veterinarians when a patient with monkeypox is suspected.

The visit should be planned so that it does not coincide with other clients and to be able to thoroughly disinfect. The suspected case should be the last one of the day.
The suspected animal should not be allowed to enter through the waiting area of a veterinarian clinic, nor should it be taken to a common treatment room. The number of staff allowed in the exam room and that come in contact with the suspected animal should be limited to as few persons as possible
Record all data to optimize traceability.
Use adequate IPE: FFP2, KN95, or FFP3 mask or equivalent, latex (or preferably vinyl) gloves, disposable gown, goggles or face screen
Disinfect the room and the material: sodium hydroxide (0.8%), sodium hypochlorite (1%), quaternary ammonium compounds, chloramine T (0.2%), iodide and phenol compounds (3%), certain detergents
Thoroughly clean and sterilize the material used. Non-disposable gowns, towels, and other material should be cleansed in a washing machine with a hot-water cycle. Chlorine bleach may be added as increased measure of safety
Waste should be disposed of according to local regulations. Bedding, cage, toys, food, or water bowls should not be disposed of into the general garbage
